# WeCoNET: a host–pathogen interactome database for deciphering crucial molecular networks of wheat-common bunt cross-talk mechanisms

**DOI:** 10.1186/s13007-022-00897-9

**Published:** 2022-06-03

**Authors:** Raghav Kataria, Rakesh Kaundal

**Affiliations:** 1 Department of Plants, Soils, and Climate, College of Agriculture and Applied Sciences, Utah State University, Logan, UT 84322 USA; 2 Bioinformatics Facility, Center for Integrated BioSystems, Utah State University, Logan, UT 84322 USA; 3 Department of Computer Science, College of Science, Utah State University, Logan, UT 84322 USA

**Keywords:** Annotations, Common bunt, Effector proteins, Interolog, Protein–protein interactions, Secretory proteins, WeCoNET

## Abstract

**Background:**

*Triticum aestivum* is the most important staple food grain of the world. In recent years, the outbreak of a major seed-borne disease, common bunt, in wheat resulted in reduced quality and quantity of the crop. The disease is caused by two fungal pathogens, *Tilletia caries* and *Tilletia laevis*, which show high similarity to each other in terms of life cycle, germination, and disease symptoms. The host–pathogen protein–protein interactions play a crucial role in initiating the disease infection mechanism as well as in plant defense responses. Due to the availability of limited information on *Tilletia* species, the elucidation of infection mechanisms is hampered.

**Results:**

We constructed a database WeCoNET (http://bioinfo.usu.edu/weconet/), providing functional annotations of the pathogen proteins and various tools to exploit host–pathogen interactions and other relevant information. The database implements a host–pathogen interactomics tool to predict protein–protein interactions, followed by network visualization, BLAST search tool, advanced ‘keywords-based’ search module, etc. Other features in the database include various functional annotations of host and pathogen proteins such as gene ontology terms, functional domains, and subcellular localization. The pathogen proteins that serve as effector and secretory proteins have also been incorporated in the database, along with their respective descriptions. Additionally, the host proteins that serve as transcription factors were predicted, and are available along with the respective transcription factor family and KEGG pathway to which they belong.

**Conclusion:**

WeCoNET is a comprehensive, efficient resource to the molecular biologists engaged in understanding the molecular mechanisms behind the common bunt infection in wheat. The data integrated into the database can also be beneficial to the breeders for the development of common bunt-resistant cultivars.

**Supplementary Information:**

The online version contains supplementary material available at 10.1186/s13007-022-00897-9.

## Background

Wheat (*Triticum aestivum* L.), an important staple grain throughout the world, is an integral source of plant-derived protein, calories, and other nutrients [1]. A recent report on wheat production data by the Food and Agriculture Organization of the United Nations estimated an adequate wheat supply with respect to the global demand (http://www.fao.org/worldfoodsituation/csdb/en/, Accessed 20 August 2021). However, due to the growing human population and changing dietary habits, the demand for wheat is increasing continuously. Despite the ample availability of wheat, there is a need to escalate wheat production, owing to losses in the yield (estimated to be around 21.5%) and reduced quality by a variety of pests and diseases. Among these, fungal diseases are considered as a major constraint to yield quality and wheat production, worldwide [2]. A major seed-borne disease in wheat, common bunt, is incited by two pathogenic fungi viz., *Tilletia caries* (synonym: *T. tritici*) and *Tilletia laevis* (synonym: *T. foetida*), which share high similarities in terms of germination, life cycle, and disease symptoms but differ in their morphology [3]. The disease is known to cause a significant reduction in grain yield and quality in the infected plants in comparison to the healthy plants in many parts of the world. During the infection mechanism, the wheat grains are replaced by the brown-black bunt balls that produce an unpleasant smell due to the presence of trimethylamine, thus the disease is also referred to as “stinking smut” [4, 5].

The interaction mechanism between host and pathogen involves gene-for-gene resistance, thus leading to the generation of a cascade of immune signals through various signaling pathways such as the jasmonic acid (JA) pathway, salicylic acid (SA) pathway, and others [6]. In the past years, significant research has been carried out to diagnose the infection mechanism of *Tilletia* species at certain stages of plant growth but due to the highly similar genetic makeup of *T. caries* and *T. laevis*, the success of molecular techniques/tools is limited [7]. Recently, 15 SNPs associated with bunt resistance were identified on chromosomes 2A, 3D, and 4A using genome-wide association study (GWAS) [8]. In another study, 123 SNPs related to resistance were found to be located on 14 chromosomes [9]. Although various chemical seed treatments are available to control the disease, these may cause negative effect on the environment, and also increase the production cost [10]. Due to sparse knowledge about the functions of *Tilletia* proteins, the crucial disease-related pathways and the respective genes involved in the pathway have not been deciphered.

The molecular interactions between host and pathogen are highly involved in infection mechanisms as well as in initiating disease-related defense signals. Therefore, the elucidation of protein–protein interactions (PPIs) using the systems biology approach helps in the understanding of various underlying disease mechanisms, and related pathways [11–14]. Further, the identification of potential host transcription factors and effector proteins in pathogens will enhance the knowledge of infection mechanisms, and lead to the development of resistant cultivars. Till now, there are no resources available to retrieve host–pathogen PPI data for common bunt disease. WeCoNET aims to provide the research community with a platform that serves as a resource of host–pathogen interactome data, along with the various functional annotations and enhanced PPI network of the proteins involved in common bunt disease. This is the first open-source, user-friendly interface for providing information for *Triticum*-*Tilletia* inter-species PPIs. The database is available for public use at http://bioinfo.usu.edu/weconet/.

## Construction and content

### Database design and architecture

**Fig. 1:**
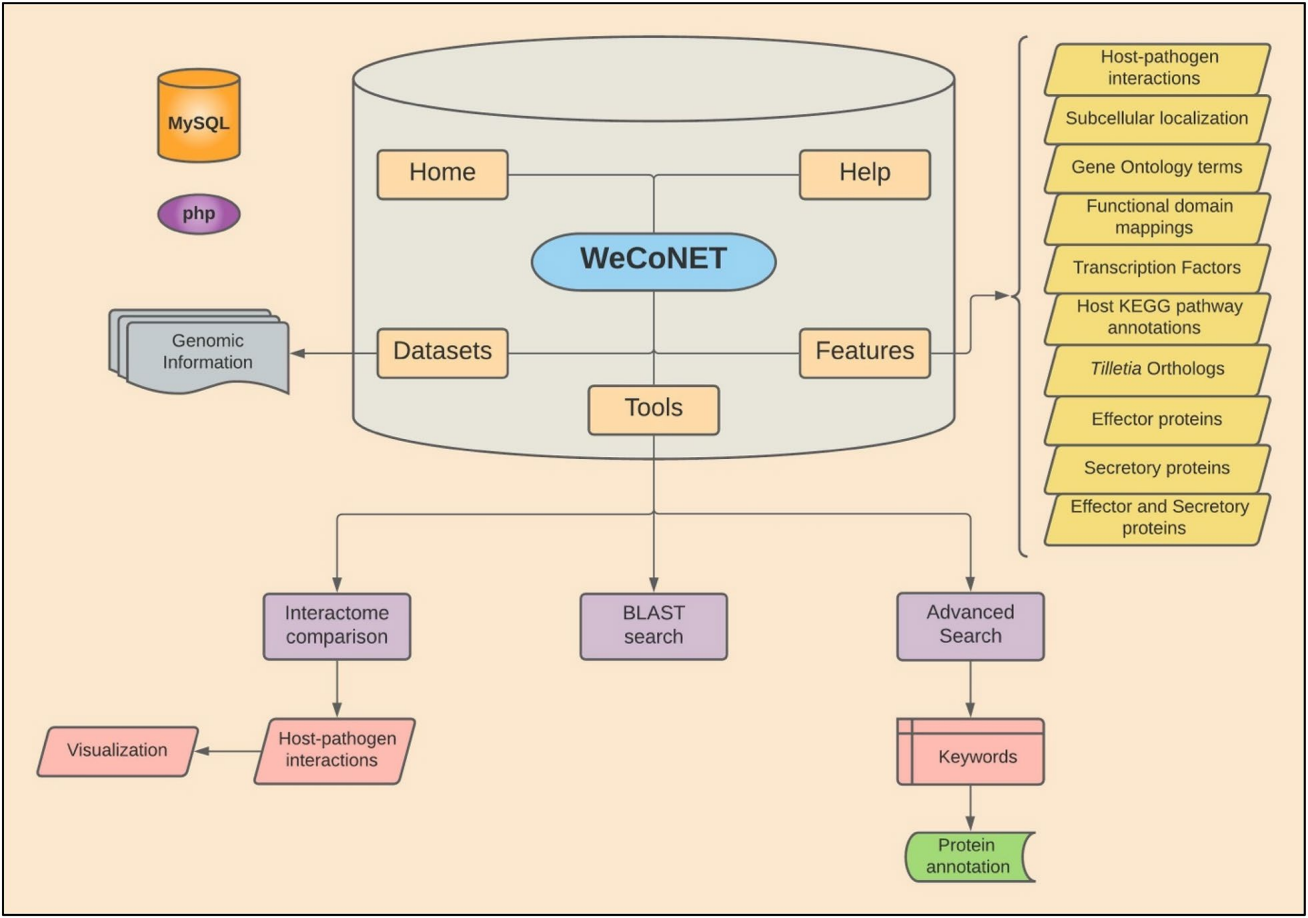
Overall framework of WeCoNET database

WeCoNET is an open-source web server, served by Apache 2.4.41. MySQL stores the data at the backend, and is queried by PHP scripts. Results are displayed via a typical JavaScript/HTML/CSS stack, and Bootstrap 4 was used as a CSS framework. WeCoNET implements a user-friendly query system that can be used to access the information of the host and pathogen protein annotations, distributed across several individual pages. To further ease the database browsing, a “Help” page is deployed, which guides the users through different tools/features of the database. The dataset for each species used to develop the database can also be accessed from the “Datasets” page. A brief overview of the database framework is available in Fig. 1**.**

### WeCoNET datasets

For the database development, the publicly available datasets of *Triticum* and *Tilletia* species were used. The proteome of each species was retrieved from their respective sources and subjected to CD-HIT [15] at 100% to avoid redundancy. The detailed information about the proteomes is available in Table 1.

**Table 1:** Sources of protein datasets used in WeCoNET

Species	Source	Number of proteins	
		Downloaded	After CD-HIT analysis
*Triticum aestivum*	Ensembl Plants (https://plants.ensembl.org/index.html, Accessed 5 April 2021)	133,346	104,701
*Tilletia caries* strain DAOM 238032	Ensembl Fungi (https://fungi.ensembl.org/index.html, Accessed 5 April 2021)	10,204	10,170
*Tilletia laevis* strain DAOMC 238040	NCBI (https://www.ncbi.nlm.nih.gov/, Accessed 5 April 2021)	9,651	9,637

### Host–pathogen interactomics tool

The host–pathogen interactome comparison tool employs sequence homology-based interolog approach, which implements seven known PPI databases, viz., HPIDB [16], MINT [17], DIP [18], STRING [19], BioGRID [20], IntAct [21], and PHI-base [22] at the backend. The proteomes of wheat and *Tilletia* species were then BLAST-searched against these PPI databases. When a user submits the job for interolog-based prediction, the resulting BLAST alignment files are subjected to in-house R scripts and various SQL queries, which are based on BLAST parameters such as identity, e-value, and coverage, as provided by the user. In this module, the network visualization for the predicted interactions is implemented using SigmaJS (http://sigmajs.org/, Accessed 11 September 2021).

### BLAST module

The BLAST module in the database implements the proteomes of wheat and *Tilletia* species at the backend. For displaying the aligned results in an interactive format, we employed a JavaScript-based library, BlasterJS [23] that provides a user-friendly exploration of the BLAST alignments.

### Annotation sources for different features

The database implements pre-calculated interolog-based host–pathogen PPIs between wheat and *Tilletia* species. These interactions are based on the highest number of effector and secretory proteins obtained from the filtered BLAST alignments (identity ≥ 30%, evalue ≤ 1e-04, coverage ≥ 40%). To obtain the “functional annotations” of wheat and *Tilletia* species proteins, we analyzed the proteins in the standalone version of InterProScan [24], which provided functional information of the proteins such as protein description, protein length, conserved domains, and essential sites. The GO terms for the proteins were also obtained using “goterms” parameter in InterProScan. The “subcellular localization” of the host proteins was obtained using the SVM-based tool, Plant-mSubP (http://bioinfo.usu.edu/Plant-mSubP/, Accessed 26 April 2021) [25], while the localization of pathogen proteins was predicted using DeepLoc 1.0 (https://services.healthtech.dtu.dk/service.php?DeepLoc-1.0, Accessed 27 April 2021) [26].

To predict the wheat proteins that serve as transcription factors, we analyzed the proteins on PlantTFDB 4.0 (http://planttfdb.gao-lab.org/, Accessed 18 August 2021), a publicly available database of transcription factors in green plants [27]. The database also implements the information about the “KEGG pathways” in which the host proteins are involved. Due to limited annotation information available for the wheat proteins, the KEGG pathways were obtained using KOBAS server [28], and in-house python scripts.

The effector proteins are generally known to hijack host cell machinery, and suppress the basal immune system [29], whereas the secretory proteins perform diverse functions such as protein translocation, acquisition of nutrients from the host, signaling, and others [30]. In this regard, we identified the proteins of *T. caries* and *T. laevis* that serve as effector and secretory proteins using the online web servers, EffectorP 2.0 (http://effectorp.csiro.au/, Accessed 23 April 2021) [31] and SignalP-5.0 (https://services.healthtech.dtu.dk/service.php?SignalP-5.0, Accessed 23 April 2021) [32], respectively. Furthermore, since *T. caries* and *T. laevis* are highly similar to each other, hence the “orthologs” of both the *Tilletia* species were predicted using OrthoFinder [33].

## Utility and discussion

In recent years, systems biology has gained a significant interest of researchers around the globe. The disease infection mechanism involves various molecular interactions, amongst which, protein–protein interactions play a crucial role. Hence, in-depth knowledge of the biological functions of the proteins provide more insights into the host defense mechanisms and pathogen infection [34]. The molecular techniques such as co-immunoprecipitation, yeast two-hybrid are suitable for the prediction of a limited number of PPIs and are time-consuming, labor-intensive. Therefore, to predict PPIs on a large-scale, computational approaches are highly efficient [35]. In the past years, the increase in the number of known PPIs has led to the development of various PPI databases [36]. WeCoNET is a reservoir of numerous functional protein annotations of wheat and *Tilletia* species proteins involved in the common bunt disease.

### WeCoNET tools

**Fig. 2:**
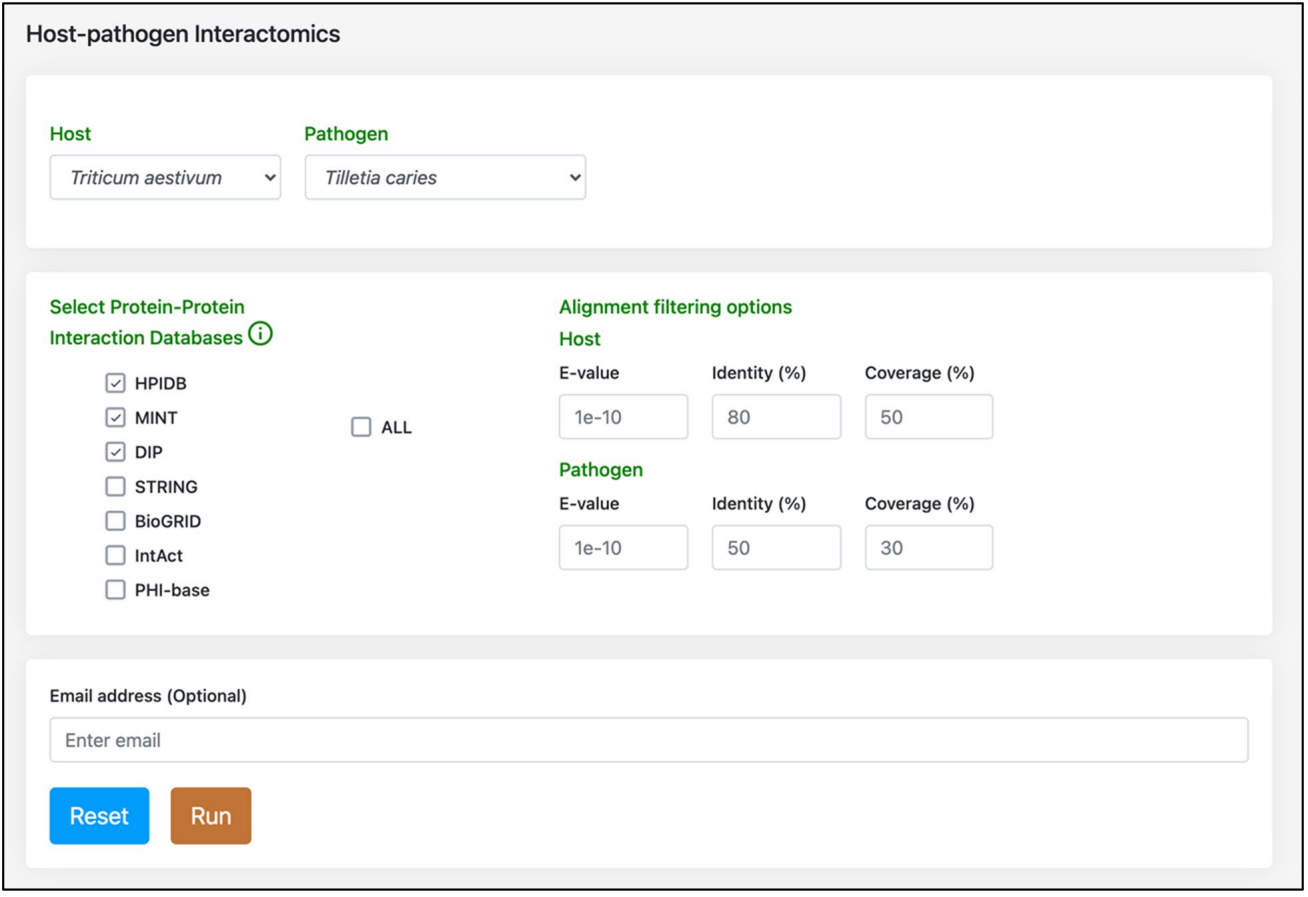
Host–pathogen interactomics tool of WeCoNET

**Fig. 3:**
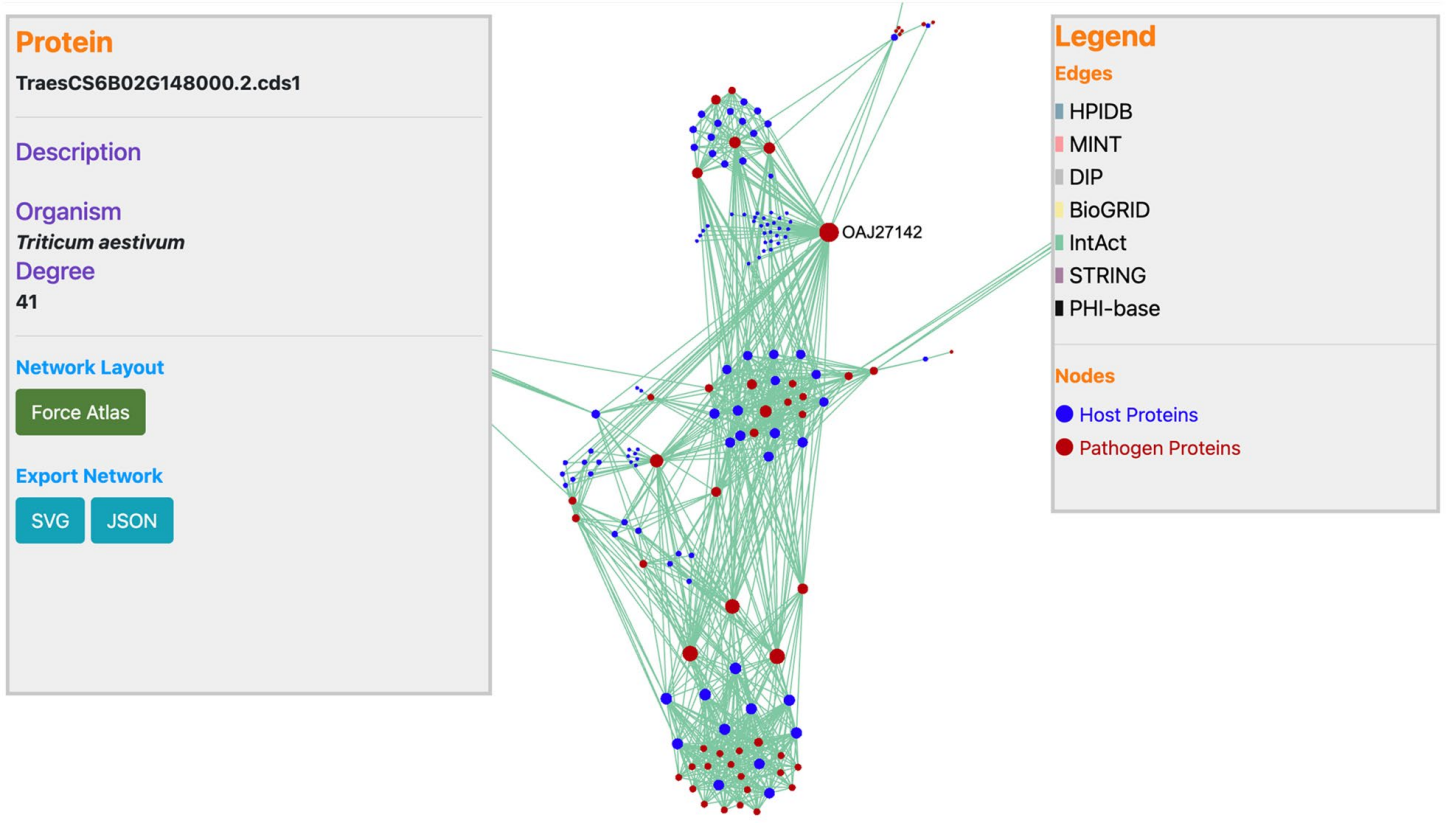
Network visualization of the predicted protein–protein interactions through the host–pathogen interactome tool

Common bunt is caused by two different species (*T. caries* and *T. laevis*), belonging to the same genus “*Tilletia*” that are highly similar to each other. Thus, a comparative study of both the pathogen strains will lead to species-specific treatment of the disease in wheat. The database implements three tools that provide information about the host–pathogen PPIs and their functional annotations. The primary functionality is the “interactome comparison” tool, whereby the user can predict interolog-based PPIs between wheat and the two *Tilletia* species (Fig. 2). The users can select the PPI databases of their choice and define customized BLAST alignment filtering options (% identity, e-value, and % coverage). The (optional) e-mail feature in the module enables the user to get alerts for the completed job. The resulting interactome is provided in downloadable (Excel or PDF) tabular format, which can also be filtered using keywords. Additionally, the network of the predicted PPIs can be visualized, which also provides the information (such as protein name, organism, description, and degree) of the proteins in the network (Fig. 3). The different colored edges in the network represent the known PPI databases selected by the user to predict the PPIs. The network can also be exported in SVG/JSON format, thus enabling the user to enhance the network in other visualization packages.

**Fig. 4:**
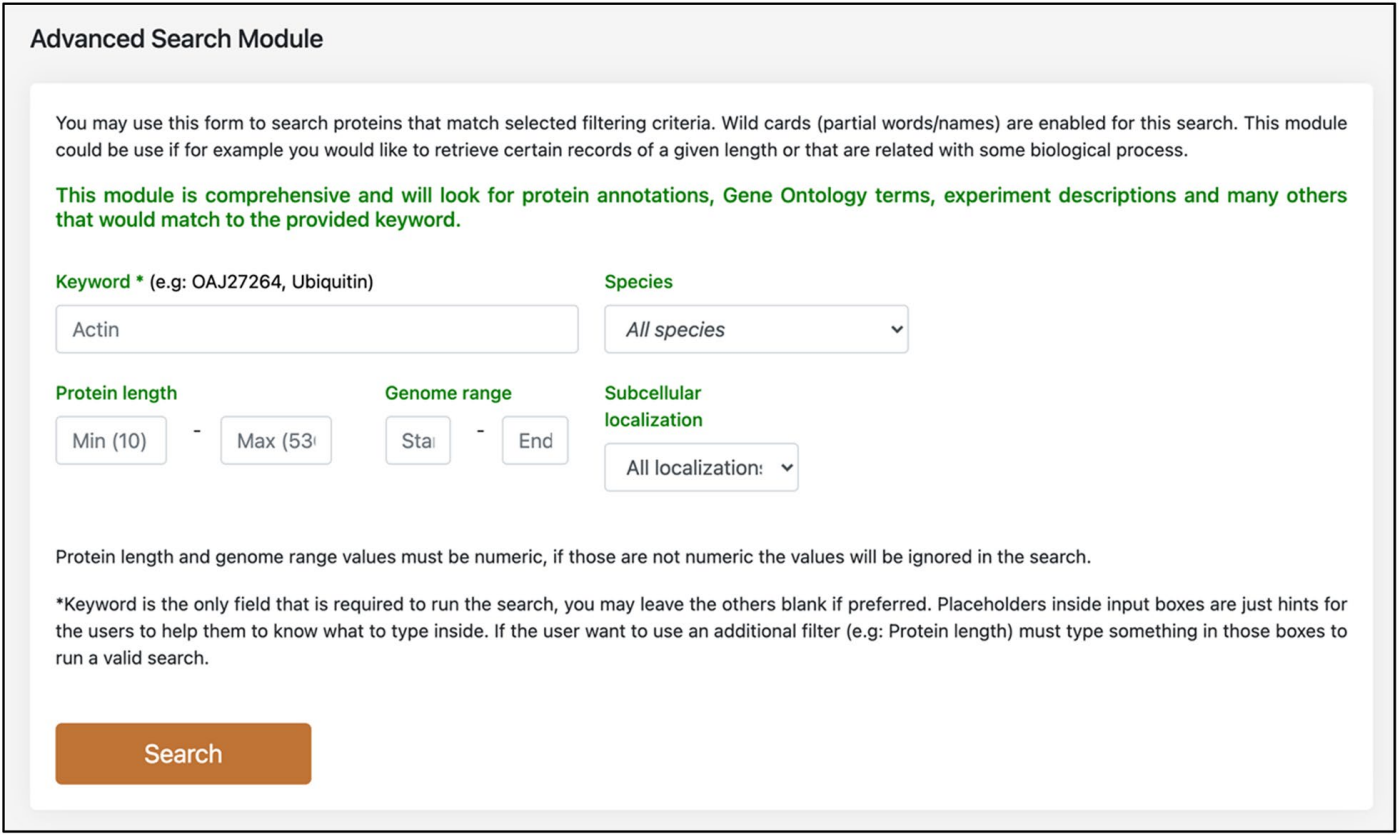
Interface of the comprehensive advanced search module

WeCoNET facilitates the users to filter the *Tilletia* proteins using keywords and/or by genomic information parameters through an extensive “advanced search” module (Fig. 4). The proteins can also be filtered according to a specific subcellular localization of the protein. Being comprehensive in nature, this module compiles functional information from various other features of the database (such as localization, functional domain mappings, InterPro annotations, gene coordinates) in accordance with the search criteria provided, thus performing several tasks/queries in a fraction of seconds. The sequences of the resultant proteins are also available. The availability of multiple features in a single interface makes the database user-friendly and more time-efficient.

**Fig. 5:**
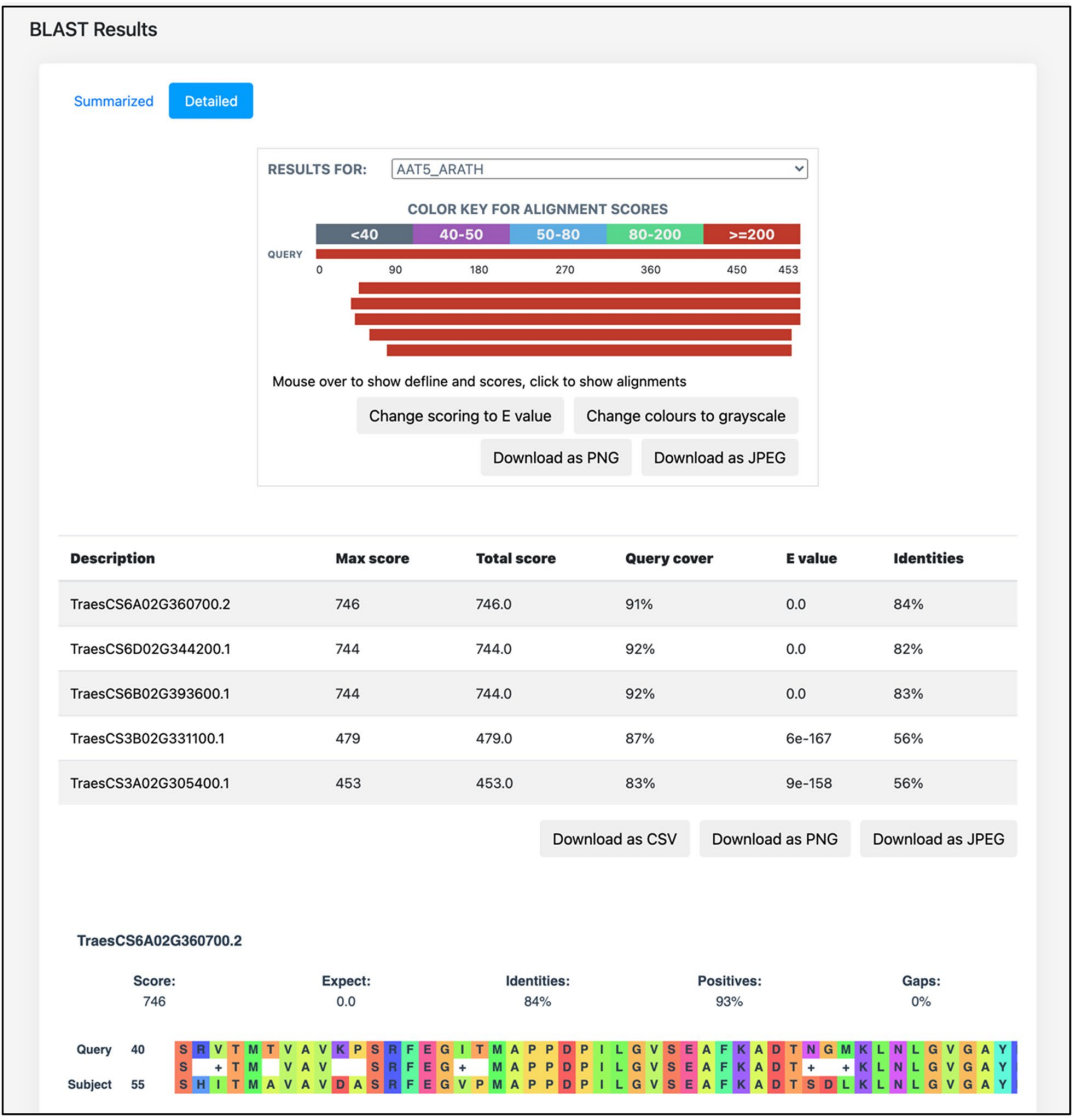
The “Detailed” submenu of BLAST alignment results page

Apart from the functional annotations, the database also hosts the “BLAST search” tool, implementing the proteomes of wheat, *T. caries*, and *T. laevis*, which can be BLAST-searched using any user-provided sequences. An optional email feature is also provided that sends a link of the BLAST alignment results to the user after completion. The tool automatically detects whether the provided sequence is a nucleotide or amino acid sequence, and performs the BLAST alignment (BLASTp or BLASTx) accordingly. The resulting alignments are available to download in Excel/PDF format. From the “Detailed” submenu on the BLAST results page (Fig. 5), the alignments can also be visualized using an enhanced graphical form that is downloadable as JPEG/PNG format. This page also allows the user to change the scoring method (Max score or e-value), and color schema (grayscale or full colored) of the BLAST alignment results.

### Functional annotations in WeCoNET

**Fig. 6:**
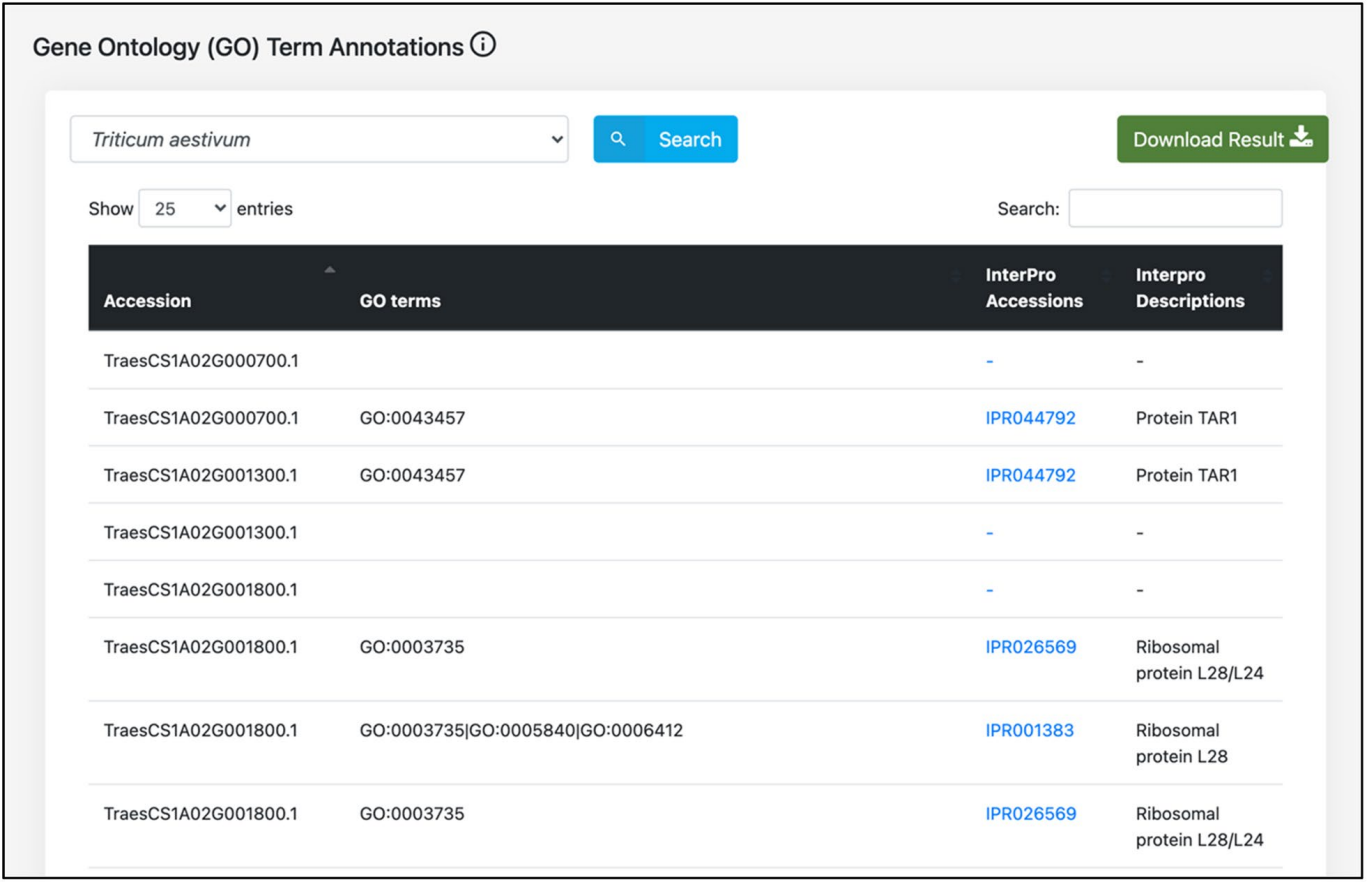
Gene ontology (GO) term annotations of the host proteins incorporated in the database

WeCoNET implements various functional annotations of *T. aestivum* and *Tilletia* species proteins under the “Features” submenu. The common functional annotations for host and pathogen proteins include subcellular localizations, gene ontology (GO) term annotations, and functional domain mappings. The gene ontology module includes the GO terms in which the proteins are involved. The InterPro accessions of the proteins have been linked to InterPro (https://www.ebi.ac.uk/interpro/, Accessed 14 July 2021) for a detailed description (Fig. 6). In the “functional domain mappings” module, various identified protein domains have been implemented, along with their respective source of prediction and description of the domain.

**Fig. 7:**
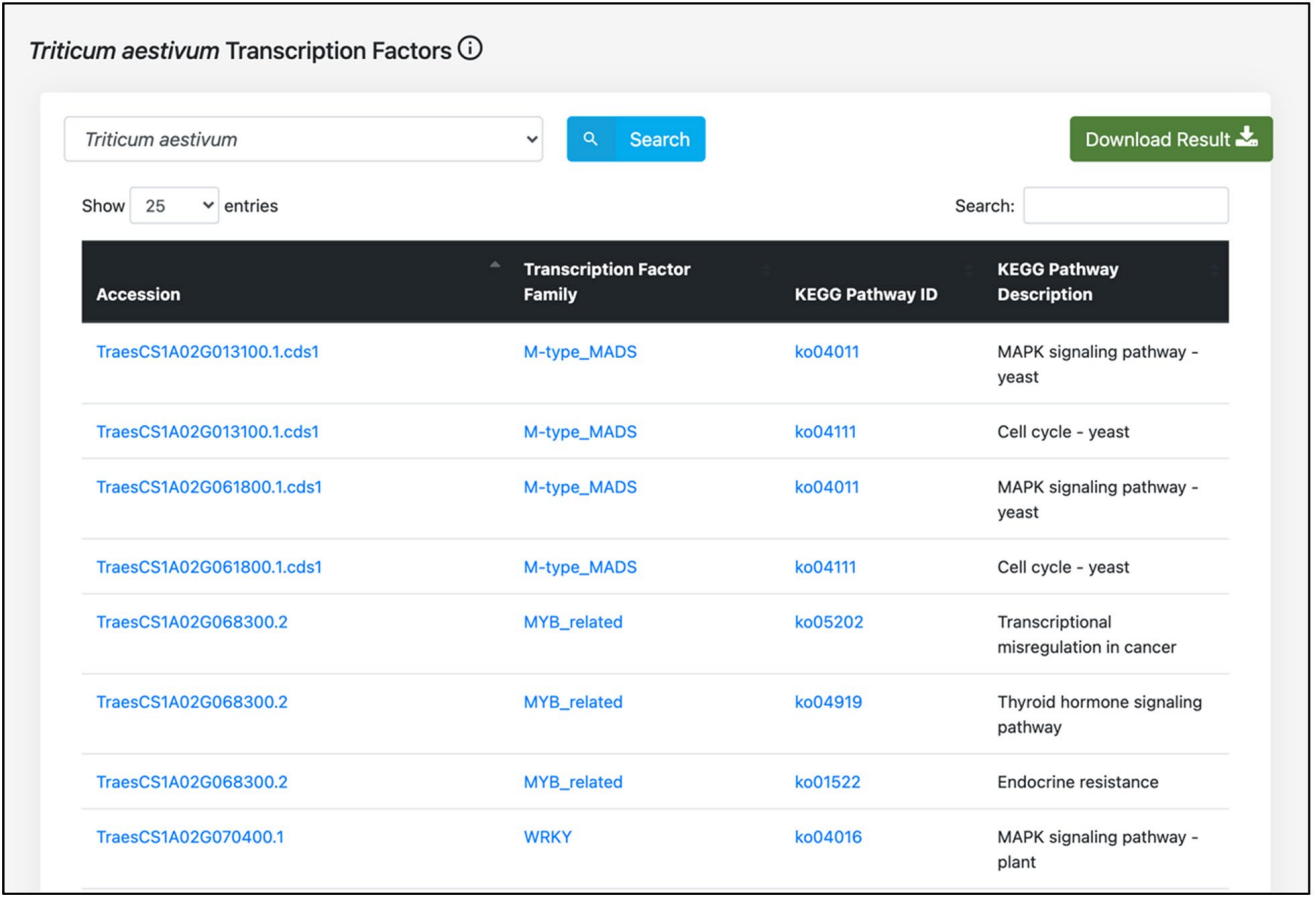
Transcription factor family, and KEGG pathway IDs of the host proteins

The host proteins that serve as transcription factors are implemented on the database, along with their respective transcription factor family and KEGG pathway, which are further linked to external databases (PlantTFDB and KEGG GENOME database, respectively) to obtain detailed information about the transcription factor family and biological pathway (Fig. 7). KEGG pathway ID and KEGG pathway description of host proteins are also available as a separate feature. To better understand the role of the host proteins in biological pathways, these proteins have been linked to respective pathways in the KEGG GENOME database.

**Fig. 8:**
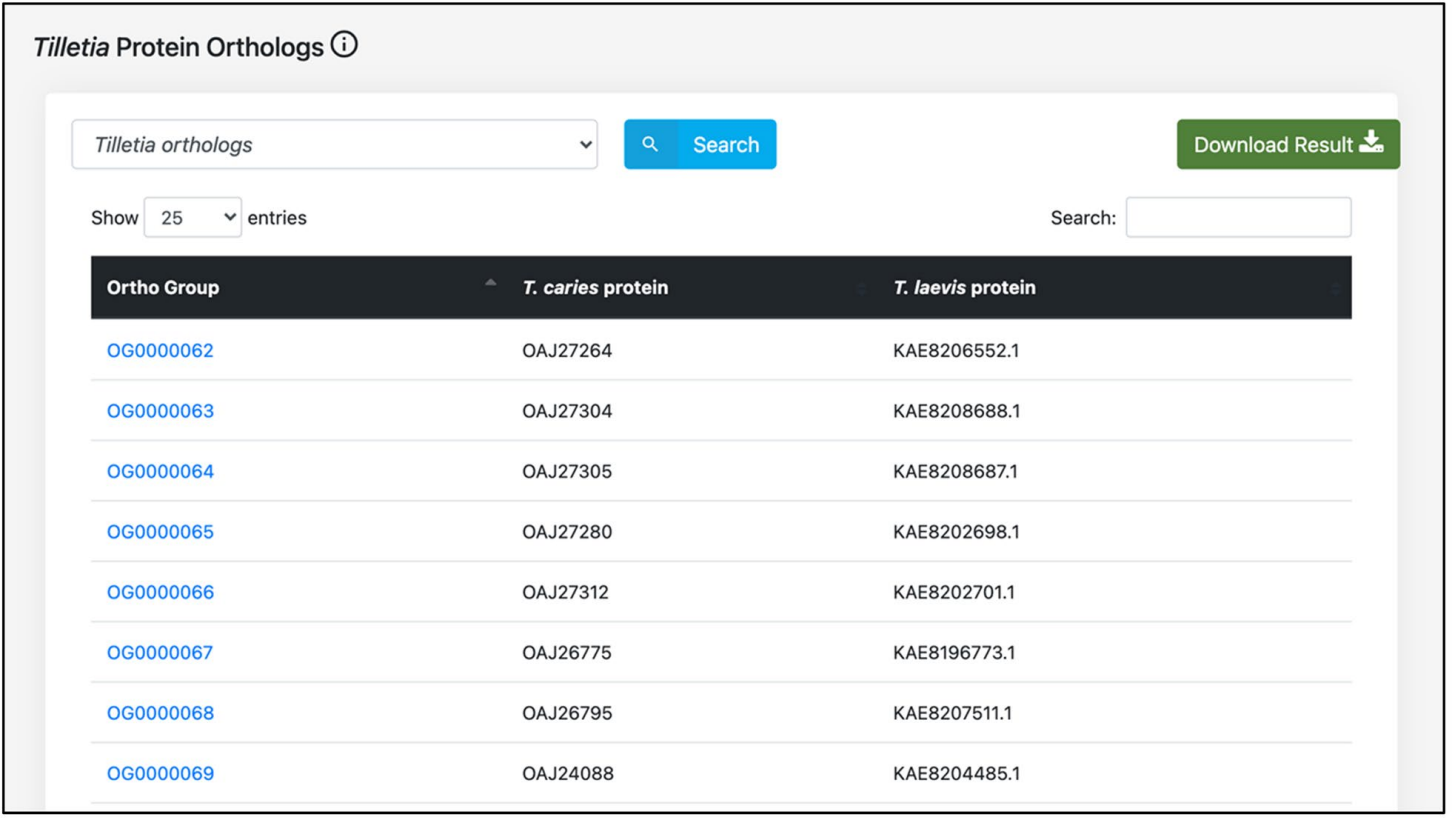
*Tilletia* species proteins predicted as Orthologs

Since both the *Tilletia* species are highly similar to each other, we decided to predict the proteins that are orthologs of each other, which led to 423 *Tilletia* orthologs. The users can download the protein sequences of the orthologs using the ‘Ortho Group’ identifier (Fig. 8). Further, the annotational information about the *Tilletia* proteins that serve as effector and secretory proteins is also available. We also identified the *Tilletia* species proteins that serve as both effector and secretory proteins, which are implemented as a separate module in the database.

### Experimental validation: a study on common bunt resistance SNPs/QTLs in *Triticum aestivum*

To validate the predicted PPIs in WeCoNET, various studies that reported the identification of common bunt resistance SNPs/QTLs in *T. aestivum* were taken into consideration [8, 9, 37, 38]. The reported SNPs/QTLs were primarily identified using genotyping-by-sequencing (GBS), and GWAS. Additionally, 16 race-specific common bunt resistance (R) genes (*Bt1-Bt15*, *Btp*) have been identified, few of which have been mapped [39]. These SNPs/QTLs/R genes were identified on different wheat chromosomes, viz., 1A, 1B, 1D, 2A, 2B, 2D, 3A, 3B, 3D, 4A, 5A, 5B, 5D, 6A, 6B, 6D, 7A, 7B, and 7D. The 72,201 host proteins associated with bunt-resistance chromosomes were found interacting with 386 pathogen proteins, which resulted in 4,219,533 interactions. These host proteins can be considered as novel targets for further understanding the protein function.

For further validation, we selected one interaction pair randomly, for example, “TraesCS1A02G323900.1—OAJ08159”, and investigated the biological function/pathway of host and pathogen proteins. The analysis showed that the host protein “TraesCS1A02G323900.1” is involved in small GTPase mediated signal transduction (GO:0007264), which is known to play a crucial role in disease resistance in rice [40, 41]. KEGG pathway enrichment revealed that this protein is also involved in biosynthesis of secondary metabolites (ko01110). Secondary metabolites are believed to be implicated in plant defense, and stress responses [42]. While the pathogen protein “OAJ08159” showed its role in GTPase activity (GO:0003924). A recent study showed the role of dynamin-like GTPase protein homologue (FgSey1) in the development and pathogenicity of a pathogenic fungus, *Fusarium graminearum* [43]. The functional analysis of the above-mentioned host–pathogen PPI pair shows it to be a candidate pair for experimental validation, thus confirming the viability of the database. Furthermore, based on a recent transcriptome analysis on wheat tassel [44], we found a few more potential PPI pairs in which the wheat genes show up- or down-regulation during the common bunt infection. These PPI pairs are available in Additional file 1.

## Future developments

Currently, WeCoNET contains functional annotations of host and pathogen proteins involved in common bunt infection mechanism. Due to inadequate annotational information of the *Tilletia* species proteins, this information will be updated with the latest annotations from experimental validations. We will also implement domain-based computational approach on the database to predict the host–pathogen PPIs. To ensure the research community with the latest information, the proteomes of wheat and *Tilletia* species used in various tools will be upgraded at the backend. The future updates may also involve the incorporation of other pathogen strains responsible for causing common bunt in wheat, thus enhancing the quality of the database.

## Conclusions

WeCoNET is a web-based, freely available host–pathogen protein–protein interaction database, which hosts functional protein annotations of *Triticum* and *Tilletia* species proteins involved in common bunt disease. Apart from the functional annotations, the database implements “interactome comparison” tool to predict interolog-based PPIs between wheat and *Tilletia* species, followed by network visualization of the PPIs. The BLAST utility enables the users to align the sequences against wheat and *Tilletia* proteomes. We believe WeCoNET would serve as an advantageous resource, and aid the research community in deeper understanding of the common bunt disease infection mechanism.

## Supplementary Information

**Additional file 1.** Potential protein-protein interaction pairs for experimental validation.

## Data Availability

WeCoNET can be freely accessed at http://bioinfo.usu.edu/weconet/, and has been tested in the browsers such as Google Chrome, and Safari.

## Abbreviations

JA: Jasmonic acid
SA: Salicylic acid
GWAS: Genome-wide association study
PPIs: Protein–protein interactions
GO: Gene ontology
GBS: Genotyping-by-sequencing
